# Aerodynamic Investigation on a Coaxial-Rotors Unmanned Aerial Vehicle of Bionic Chinese Parasol Seed

**DOI:** 10.3390/biomimetics9070403

**Published:** 2024-07-02

**Authors:** Wenbiao Gan, Yunpeng Wang, Hongbo Wang, Junjie Zhuang

**Affiliations:** 1 Institute of Unmanned System Research, Beihang University, Beijing 100191, China; 2 School of Aeronautic Science and Engineering, Beihang University, Beijing 100191, China; wangyunpeng02@buaa.edu.cn (Y.W.); 19377094@buaa.edu.cn (J.Z.); 3 Innovation and Application Center, China Academy of Aerospace Aerodynamics, Beijing 100074, China

**Keywords:** aerodynamic investigation, coaxial rotors, bionic design, numerical simulation, experimental measurement

## Abstract

Aerodynamic investigation of a bionic coaxial-rotors unmanned aerial vehicle (UAV) is performed. According to Chinese parasol seed features and flight requirements, the bionic conceptual design of a coaxial-rotors UAV is described. A solution procedure for the numerical simulation method, based on a multi-reference frame (MRF) model, is expressed, and a verification study is presented using the typical case. The aerodynamic design is conducted for airfoil, blade, and coaxial-rotors interference. The aerodynamic performance of the coaxial rotors is investigated by numerical simulation analysis. The rotor/motor integrated experiment verification is conducted to assess the performance of the coaxial-rotors UAV. The results indicate that the UAV has excellent aerodynamic performance and bionic configuration, allowing it to adapt to task requirements. The bionic UAV has a good cruise power load reach of 8.36 kg/kw, and the cruise flying thrust force is not less than 78 N at coaxial-rotor and rotor-balloon distance ratios of 0.39 and 1.12, respectively. It has the “blocks stability phenomenon” formed by the rotor downwash speed decreases and the balloon’s additional negative pressure. The present method and the bionic configuration provide a feasible design and analysis strategy for coaxial-rotors UAVs.

## 1. Introduction

As an unconventional vertical take-off and landing (VTOL) aircraft, the coaxial-rotor unmanned aerial vehicle (UAV) has the advantages of a compact structure, counter-torque mutual cancellation of the upper and lower rotors, and good hover stability and handling. It has excellent application prospects in civil and military fields [1,2,3].

Coaxial rotors have fewer structural components and higher power system efficiency than conventional multi-rotors. Still, uncertainties and irrationality exist in the mission payload compartment arrangement, particularly difficulties meeting omnidirectional detection and reconnaissance loading requirements. A coaxial-rotor UAV must consider flight stability and efficiency to arrange the mission payload compartment. It is essential to design highly efficient mission functional payload compartments at specific locations above and below the rotors, ensuring they are positioned sufficiently far away from them. This results in the formation of a combined configuration of coaxial rotors and mission functional modules.

The need for a novel configuration design incorporating coaxial rotors and a mission function module is inspired by the natural biological flight observed in the dispersal of tree seeds, which exhibits similarities to rotary flight with coaxial rotors and yaw lobes acting as rudders. In the process of drifting, the lobes of the Chinese parasol seeds rotate similarly to coaxial rotors, and the fruit joins the lobes’ roots to act as flight stabilization and to carry the fruit, similar to the mission function module. Therefore, we can use the Chinese parasol seeds for reference to develop the coaxial-rotors UAV configuration, which has coaxial rotors and mission function modules.

The coaxial rotors can eliminate the power loss caused by tail rotor interference. However, due to the compact configuration of the upper and lower coaxial rotors, a significant portion of the lower rotor area is affected by the downwash flow and wake vortex interference from the upper rotor, resulting in asymmetric interference between the upper and lower rotors, leading to more complex aerodynamic disturbances within the flow field [4]. Therefore, a coaxial rotor is less efficient than a single main rotor helicopter due to the upper-to-lower rotor–rotor interference. To fully enhance the aerodynamic performance and omnidirectional mission capability of the coaxial rotors and reduce unnecessary power consumption, it is necessary to conduct aerodynamic design and analysis studies for coaxial-rotor UAVs.

Lakshminarayan et al. [5,6] have designed and calculated miniature coaxial rotors, focusing on aerodynamic interference between the coaxial rotors at different rotor pitches and rotational speeds. They obtained clear and intuitive wake boundaries for miniature coaxial rotors. Based on the Navier–Stokes equation and the established free wake model for coaxial rotors, the numerical method of aerodynamic interference analysis was developed, and the unsteady aerodynamic interference characteristics of coaxial rotors were studied and analyzed [7,8,9]. Lei et al. [10] have employed the sliding mesh method to analyze the aerodynamic performance of small coaxial rotors against wind interference under natural incoming flow. In terms of the loading capacity of coaxial rotors, the aerodynamic performance of single and coaxial rotors was studied by hovering force test and numerical simulation of coaxial rotors, proving that coaxial rotors can improve the lift deficiency of single rotors under specific circumstances [11,12,13]. Ma al. [14,15] used particle image velocimetry (PIV) to study aerodynamic interference characteristics of coaxial rotors flow fields in different conditions.

These studies provide many references for the aerodynamic design and analysis of coaxial rotors. However, the intuitive representation of aerodynamic research on coaxial rotors still has the following deficiencies:

(1) There is still relatively limited research on coaxial-rotor configuration optimization and overall aerodynamic performance analysis [16];
(2) The results are unitary, and less attention is paid to designing and analyzing new efficient bionics that emulate nature.
(3) Due to limited experimental and computational resources, it is necessary to conduct comparative validation between calculations and experiments based on design requirements to reflect the accuracy and practicality of simulation results. The MRF (Moving Reference Frame) model used is suitable for steady-state calculations, employing a fixed rotating reference frame to handle the flow in rotating regions.

Therefore, this paper examines the aerodynamic features of a bionic coaxial-rotors unmanned aerial vehicle (UAV). According to Chinese parasol seed features and UAV flight requirements, the bionic conceptual design is described. A solution procedure for the numerical simulation method, based on multi-reference frame (MRF) model, is expressed, and a verification study is presented using a typical case, yielding favorable results. The aerodynamic design was conducted for airfoil, blade, and coaxial-rotor interference. The aerodynamic performance of the coaxial rotors was investigated by numerical simulation analysis. Comparisons were made with the performance of a single-rotor UAV. The rotor/motor integrated experiment verification was conducted to assess the performance of the coaxial-rotor UAV. The sections of this paper include Conceptual Bionic Design, Numerical Simulation Method, Aerodynamic Design, Performance Investigation, Experimental Verification, Key Results, and Feasible Design Strategy.

## 2. Conceptual Bionic Design of UAVs

In nature, seeds of some plants can drift with the wind to achieve seeding, and the Chinese parasol seed has the typical mode of long-duration drifting seeding [17,18,19]. The seed of Chinese parasol is composed of fruit and several lobes, which rotate with the wind when floating and have highly efficient coaxial-rotor flight characteristics [20,21]. This can inspire the design of coaxial-rotor UAVs [22,23].

### 2.1. The Design Requirement of a Coaxial-Rotor UAV

The coaxial-rotor UAV design requirements can be refined based on the the Chinese parasol seed, combined with mission capabilities such as aerial detection, surveillance, etc. Specific requirements are:

(1) In terms of efficiency, a coaxial-rotor power system is adopted to enhance lift and power payload efficiency. It results in higher cruise efficiency within a specific altitude range of 0–5 km.
(2) Regarding stability, the coaxial rotors’ power system, consisting of upper and lower motors and folding rotors, should achieve balanced torque and variable pitch control during a flight in the 0–5 km altitude range, despite the yaw-torque authority being less than that of a conventional single main rotor helicopter.
(3) In terms of mission payload, a downward-mounted spherical package payload compartment is employed as far as possible. It allows the center of mass to move down for UAV stability and provides partial buoyancy to achieve omnidirectional detection and surveillance.

Therefore, the coaxial-rotor UAV has two sets of foldable motor rotor power components, which are coaxially connected and can have individual pitch variation. The lower component is connected to a balloon-shaped load compartment with folding rotors and retractable balloons.

According to the design requirements of the UAV, specific constraints can be identified for the aerodynamic design of the coaxial rotors system. The optimal operating state for cruising is determined after considering the quadratic relationship between rotational speed and throttle at 60% throttle. The rotational speed of the optional 35-inch rotor with coaxial rotors/power should be kept below 2235 rpm. A lower cruise rotational speed is preferable for optimal performance [24]. Therefore, the rotor takes an equivalent size of no more than 35 inches and should be designed to achieve sufficient pull and higher force efficiency at the lowest possible rotational speed.

### 2.2. Bionic Design Modeling of the Coaxial-Rotors UAV

Inspired by the floating behavior of Chinese parasol seeds in nature, a biomimetic concept design for a coaxial-rotor UAV is developed. Chinese parasol seeds serve as the biomimetic model for the design of the coaxial-rotor UAV [25]. The characteristics of the Chinese parasol seed flight process are as follows: (1) The fruit is spherical, and the lobes fold towards the direction of the fruit. The seed-shedding flight starts, forming a tube-like whole. (2) After falling off, the seed lobes deploy, generally four lobes, and divide into two diagonal groups. Each group has a certain amount of misalignment, with two groups of lobes rotating and falling. (3) When descending to landing, the shape of the two groups of lobes of the seeds turn outward with decreasing altitude, and the rotation speed increases. At the same time, the seeds have some wind resistance stability and could land in a fruit touchdown attitude. Based on these flight characteristics of the Chinese parasol seeds, the UAV bionic conceptual design can be divided into three designs: folding cylinder type, initiated flyby, and variable altitude variable speed flight [26,27].

#### 2.2.1. Three-Dimensional Tube Folding Design

**Figure 1:**
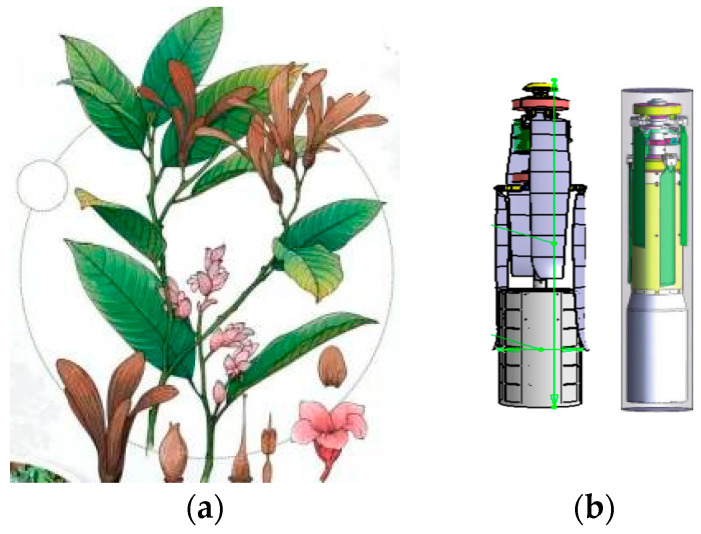
Modeling sketch of a three-dimensional tube folding design. (**a**) Structure diagram of Chinese parasol; (**b**) three-dimensional tube folding state.

Referring to the maturation process of Chinese parasol seeds and observing the shape of the lobe folding package, a three-dimensional cylindrical folding design was applied to the UAV (as shown in the diagram in Figure 1, with lobes folded and wrapped in shape as seen in the left second seed above).

#### 2.2.2. Initiated Flyby Configuration Design

**Figure 2:**
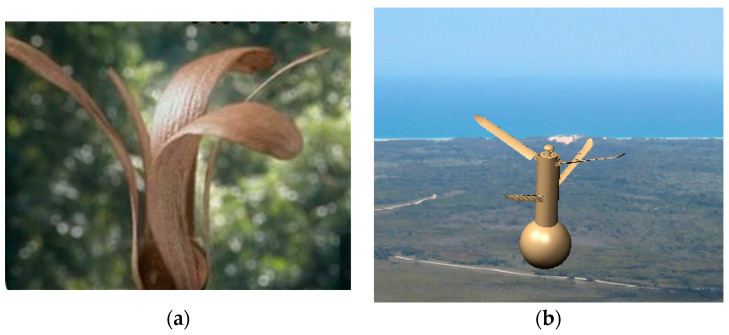
Design modeling sketch of the initiated flyby configuration. (**a**) Instantaneous morphology of Chinese parasol seed shedding; (**b**) initiated flyby configuration.

When starting the drift, the UAV rotates and deploys the coaxial rotors. This action is inspired by the flying form of detached seeds of Chinese parasol, transitioning from starting to cruising while meeting the design requirements. At this point, the rotation speed of the coaxial rotors decreases, and the payload compartment of the balloon moves downward and inflates. The three-dimensional cylindrical structure forms a whole with the balloon and coaxial rotors, similar to when the leaves of a Chinese parasol seed fall off and rotate [28]. Subsequently, as the height decreases, the cylindrical structure will gradually detach from the balloon payload compartment. Therefore, the UAV can draw on the instantaneous morphology of Chinese parasol seed shedding to design an initiated flyby configuration (as shown in Figure 2).

#### 2.2.3. Variable Altitude Variable Speed Flight Configuration Design

**Figure 3:**
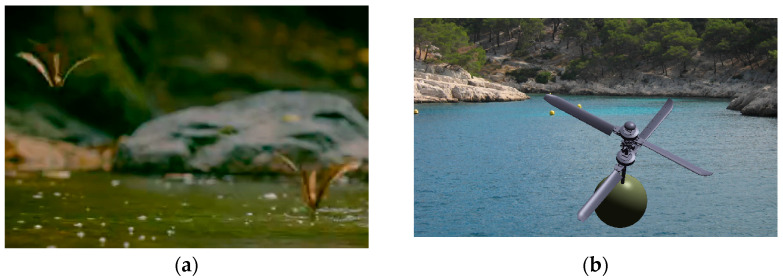
Modeling sketch of variable altitude variable speed configuration design. (**a**) Chinese parasol seed descent flight; (**b**) modeling configuration for altitude-variable speed flight.

For the variable altitude flight phase, the UAV combines the multiple forms and accelerated rotation characteristics of the Chinese parasol seed descent flight to achieve high efficiency and sufficient stability. The cylindrical structure of the UAV detaches, and the coaxial rotors fully deploy. The rotor blade pitch varies to accommodate the demands of flight lift and stability. A modeling configuration for altitude-variable speed flight (as depicted in Figure 3) can be designed accordingly.

**Figure 4:**
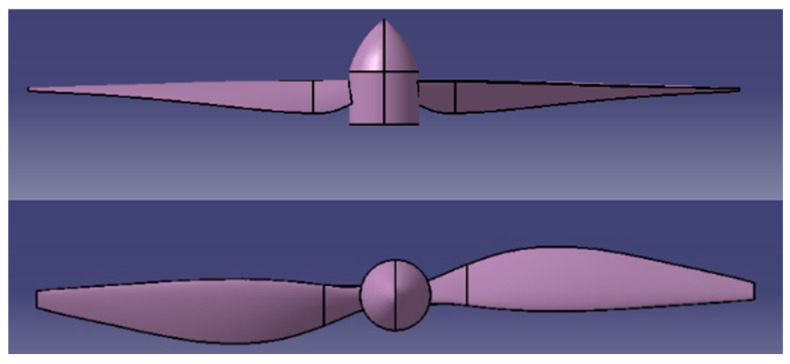
Initial design outline sketch of the rotor.

Based on design requirements and bionic design modeling, Figure 4 provides an initial design sketch of the rotor [29]. The blade’s initial airfoil shape adopts the Clark-Y airfoil profile. The installation angle distribution from root to tip of the blade is determined based on the optimal effective angle of attack of each airfoil profile along the blade.

Considering the need for comparative design analysis of the coaxial rotors, the distance ratios between the upper and lower rotors, and between the lower rotor and the balloon, are defined as:

(1) $$i_{1}=\frac{D_{1}}{R}$$

(2) $$i_{2}=\frac{D_{2}}{R}$$

*D*_1_ and *D*_2_ are the distances between the upper and lower rotors, and between the lower rotor and the balloon, respectively. *R* is the rotor radius [30].

## 3. The Numerical Method

### 3.1. The Numerical Method

**Figure 5:**
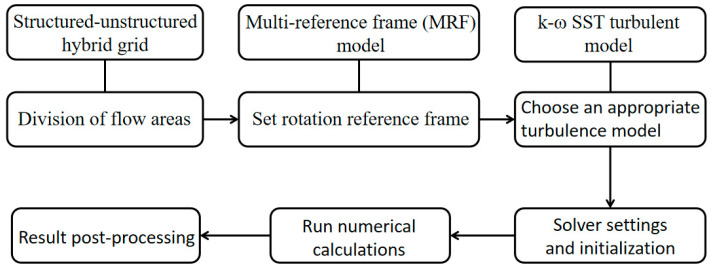
Schematic diagram of numerical scheme.

This paper employs the multi-reference frame (MRF) model combined with structured-unstructured hybrid grid techniques based on the SST turbulence model. It aims to provide a quasi-steady solution of the Reynolds mean Navier–Stokes (RANS) equation. The spatial discretization method adopts the second-order upwind MUSCL (Monotone Upstream-centered Scheme for Conservation Laws) interpolation in Roe format. For time discretization and advancement, the implicit LU-SGS method is utilized.The specific calculation process is shown in Figure 5.

The MRF model method is a mathematical method for quasi-steady numerical simulation of coaxial rotors. Compared with the unsteady solution method, which consumes excessive computational resources, the MRF method is widely used in the aerodynamic calculation of fixed-axis rotating bodies. It can still obtain higher numerical simulation accuracy while saving computational resources. The main idea of the MRF model method is to simulate a propeller’s rotating motion by establishing a regular closed cylindrical flow region around the rotor. A numerical simulation of the flow field containing the rotating airflow on a static grid is achieved to establish a rotating coordinate system with the same rotating motion as the propeller. Corresponding mathematical transformations and data interpolation transfer between the rotating and non-rotating regions achieve this.

Considering the complex geometric characteristics of the highly twisted rotor blade, a structured/unstructured hybrid grid generation method is employed during the rotor grid partitioning process. That is, in the rotating basin around the rotor, the unstructured grid is divided to improve the efficiency of grid generation. A structured grid is split into a stationary domain between the rotation domain and the far field to reduce the amount of grid computation. Flow field information transfers between the rotating and stationary basins by establishing a grade separation interface [31]. Regarding the setting of the boundary conditions of the MRF method, a pressure-far-field boundary condition is applied on the boundary of the stationary domain, and the propeller or rotor in the rotation domain is specified as a no-slip wall. A pair of overlapping cylindrical closed surfaces, where the stationary domain and the rotating domain share the same boundary, are specified as interface to exchange the flow information.

The Reynolds stress-based and separation-based ideas modify Menter’s *k-ω* SST turbulent model. The transport equations of the model are summarized as follows:

(3) $$\begin{array}{l} \frac{\partial \left(\rho k\right)}{\partial t}+\frac{\partial (\rho u_{j}k)}{\partial x_{j}}=P_{k}-\beta^{\prime }\rho k\omega \\ \;\;\;\;\;+\frac{\partial }{\partial x_{j}}\left[\left(\mu +\frac{\mu_{T}}{\sigma_{k}}\right)\frac{\partial k}{\partial x_{j}}\right] \end{array}$$

(4) $$\begin{array}{l} \frac{\partial \left(\rho \omega \right)}{\partial t}+\frac{\partial (\rho u_{j}\omega )}{\partial x_{j}}=P_{\omega }-\beta \rho \omega^{2}\\ \;\;\;\;\;\;+\frac{\partial }{\partial x_{j}}\left[\left(\mu +\frac{\mu_{T}}{\sigma_{k}}\right)\frac{\partial \omega }{\partial x_{j}}\right]\\ \;\;\;\;\;\;+2(1-F_{1})\frac{\rho }{\sigma_{\omega_{2}}\omega }\frac{\partial k}{\partial x_{j}}\frac{\partial \omega }{\partial x_{j}} \end{array}$$

### 3.2. The Numerical Verification

To validate the capability of the CFD calculation method in capturing rotor aerodynamic details, wind tunnel force and pressure measurements of the rotor were utilized based on relevant literature [32]. This was done to verify the accuracy of the numerical simulation method.

**Figure 6:**
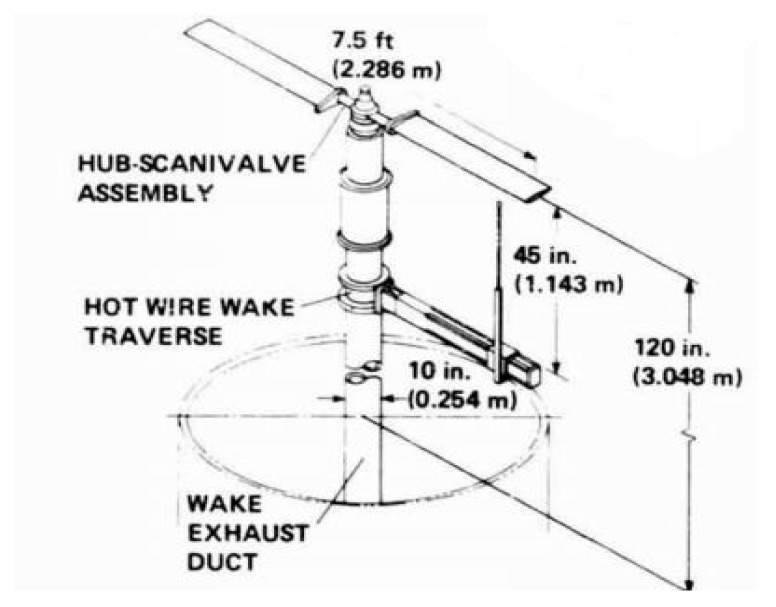
An experimental model of the rotor.

**Figure 7:**
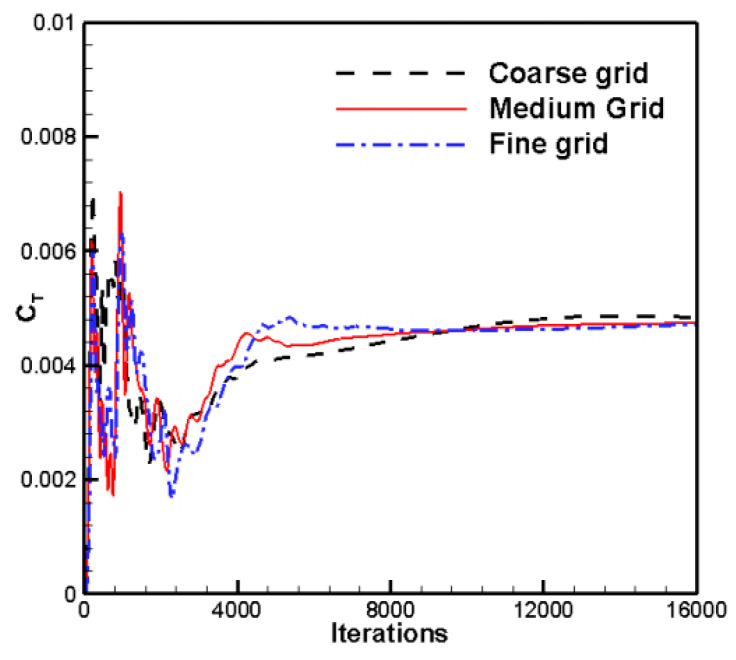
Comparison of convergence process of thrust coefficient curves for different mesh densities.

An experimental model of the rotor is shown in Figure 6. The rotor section airfoil is NACA0012, with a rotor diameter of 2.286 m and an aspect ratio of six for each blade. Experimental conditions of an 8° blade installation angle are selected for the calculation: 1750 rpm and 2250 rpm. The experimental static pressure is 103,027 Pa, temperature 289.75 K, and density 1.2389 kg/m^3^. The calculation conditions are consistent with the experimental conditions. The grid convergence testing is conducted by using three mesh densities: a coarse grid of 3.7 million cells, a medium grid of 7.5 million cells (about 5 million cells in the stationary domain and 2.5 million cells in the rotating domain), and a fine grid of 15 million cells. The corresponding y+ values for the three different grid densities are 5.1 and 0.2, respectively. A comparison of the convergence process of the thrust coefficient for different mesh densities is given in Figure 7. It is obvious that the results of the medium and fine meshes are very close, but the time consumed with the medium mesh is lower than that with the fine mesh.

**Figure 8:**
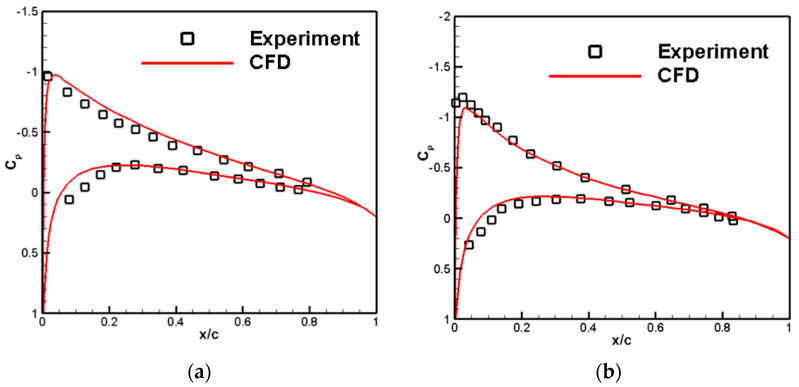
Pressure distribution at different radial sections of the rotor. (**a**) r/R = 0.68; (**b**) r/R = 0.89.

Table 1 compares rotor thrust coefficient calculations, and Figure 8 compares the chord pressure distribution for different radial position profiles of the blades. Overall, the thrust coefficient and pressure distribution calculated by CFD agree well with experimental results. Thus, the multi-reference calculation method adopted in this report demonstrates a strong capability. It effectively solves the macro aerodynamic forces and pressure distribution of rotating components.

**Figure 9:**
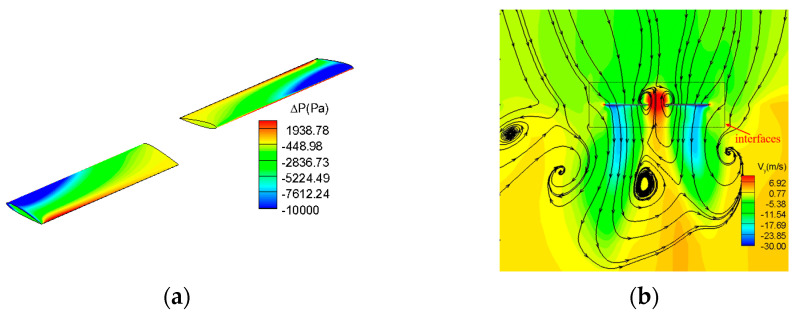
The typical flow field. (**a**) The surface pressure distribution. (**b**) The velocity distribution in the Y direction.

The typical flow field is shown in Figure 9. According to the surface pressure distribution, the present method can characterize the main flow field characteristics of the rotor surface along the spanwise direction. From the velocity distribution in the Y direction, it can be seen that the two blades induce a slight axial asymmetry. This is due to the significant unsteady characteristics of the generation, development, and diffusion of blade tip vortices in practice. Based on the Reynolds averaged equation, the multiple reference frame calculation method is used to solve the flow field of the rotor. This approach enhances computational efficiency while capturing the main flow field characteristic. It is further proved that the calculation method has comprehensive advantages in balancing calculation efficiency and precision.

## 4. The Aerodynamic Design of a Coaxial-Rotor UAV

### 4.1. Rotor Airfoil Design

**Figure 10:**
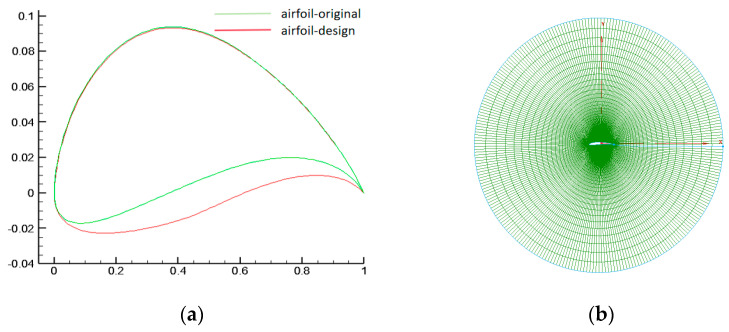
Airfoil and grid schematics. (**a**) Comparison of original and design airfoils. (**b**) Solution grid for airfoil.

For coaxial rotors in unmanned aerial vehicles, the upper and lower rotors adopt the same airfoil, divided into inner and outer airfoils in this paper. By optimizing the different designs of the upper and lower rotors, the efficiency of the total coaxial-rotor system can be further improved [33]. This consideration meets the requirements of balanced torque and variable pitch control. The parameterized design of the airfoil adopts an algebraic method based on the Hicks–Henne function. This approach effectively achieves a smooth shape and facilitates local adjustment optimization design. Figure 10 shows a schematic diagram of the original and designed airfoils and their grids.

**Figure 11:**
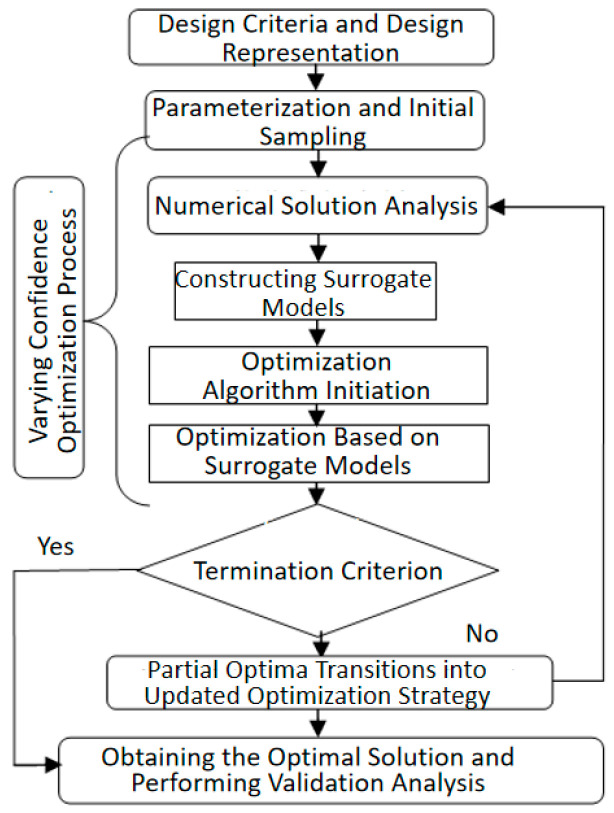
Basic Optimization Process.

In the optimization design process, the basic optimization workflow based on surrogate models is adopted, as shown in Figure 11. The optimization process includes the following parts: (1) Parametric representation and initial sampling. (2) Solving with varying levels of confidence. (3) Optimization based on the surrogate model. (4) Adding new sample points to continue optimization. (5) Validation of the design results. Detailed parameters for each level of the optimization process will be provided in the design results analysis section.

It is particularly worth noting that when conducting optimization based on surrogate models, the two-dimensional design generally employs the Kriging surrogate model, while the three-dimensional design often uses the Radial Basis Function (RBF) as the surrogate model. The optimization algorithm used is the Multi-Objective Immune Genetic Algorithm [34].

#### 4.1.1. Inner Section Airfoil

**Figure 12:**
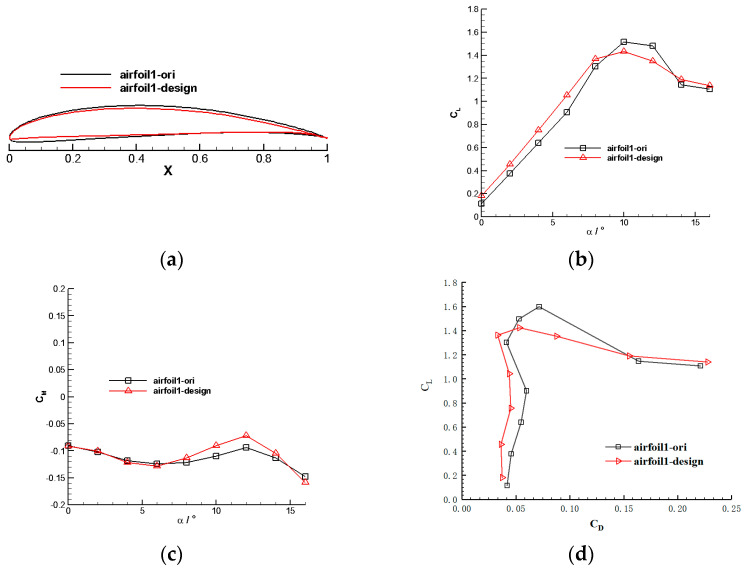
Aerodynamic characteristics of the inner rotor blade airfoil. (**a**) Optimized inner section airfoil profile. (**b**) Inner section airfoil’s lift coefficient. (**c**) Inner section airfoil’s moment coefficient. (**d**) Inner section airfoil’s lift-to-drag ratio.

Figure 12 compares the results of the inner section airfoil design. It illustrates a significant increase in the inner section airfoil’s lift-to-drag ratio over the range of available lift coefficients.

#### 4.1.2. Outer Section Airfoil

**Figure 13:**
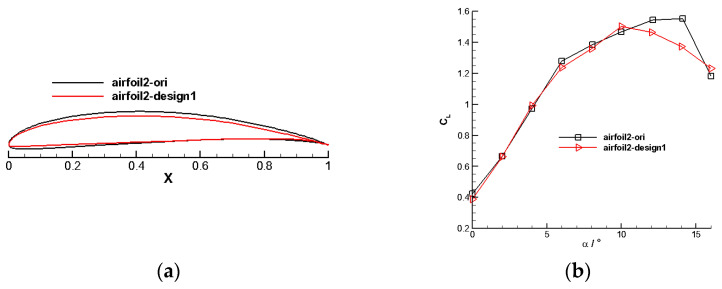

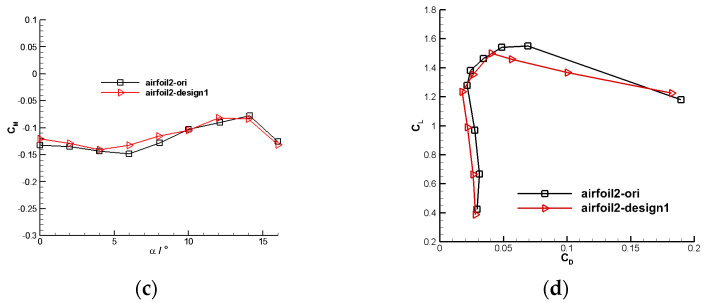
Aerodynamic characteristics of the outer rotor blade airfoil. (**a**) Optimized outer section airfoil profile. (**b**) Outer section airfoil’s lift coefficient. (**c**) Outer section airfoil’s moment coefficient. (**d**) Outer section airfoil’s lift-to-drag ratio.

The results of the outer section airfoil design are displayed in Figure 13. The figure illustrates a significant increase in the lift-to-drag ratio of the outer section airfoil under typical conditions. At the lift coefficient of 1.2, the lift-to-drag ratio of the initial airfoil (airfoil2-ori) is 53.499, while the optimized airfoil (airfoil2-design1) has a lift-to-drag ratio of 66.52, which is an improvement of 24.34% compared with the previous one.

### 4.2. The Coaxial Rotors Design

**Figure 14:**
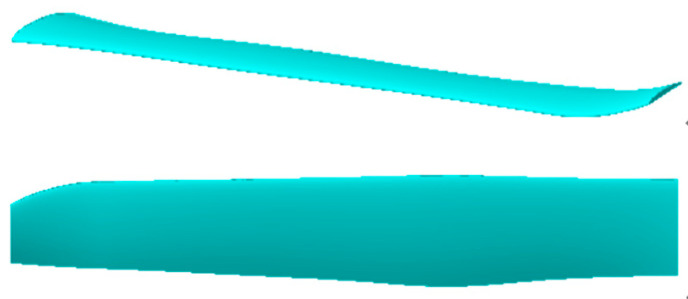
Optimized rotor blade.

Based on the airfoil design, the rotor design proceeds with different airfoils used for the inner and outer sections of the rotor blades. The rotor airfoil configuration is illustrated in Figure 14, where the “Airfoil-1” airfoil is employed for the inner section of the blades, and the “Airfoil-2” airfoil is utilized for the outer section of the blades.

The effect of blade section mounting angles is further considered for the rotor geometry. The blade radius is R = 450 mm; the chord varied progressively along the span, and there is a winglet design at the tip. Here are the specific steps of the design. The design work for this three-dimensional rotor is divided into three steps: two-dimensional airfoil optimization, airfoil configuration, and three-dimensional blade geometry optimization. The airfoil optimization aims to improve the lift-to-drag ratio to increase lift while reducing rotor torque. Based on the results of the previous chapter’s airfoil optimization, two optimized airfoils, Airfoil-1 and Airfoil-2, are used for the blade design. Airfoil-1 is applied to the inner blade section (r/R = 0.17~0.5), while Airfoil-2 is used for the outer blade section (r/R = 0.5–1.0). Building upon these first two steps, further optimization of the three-dimensional blade geometry includes iterative design of blade profile installation angles and local chord lengths. Design constraints include a maximum blade radius of R ≤ 450 mm, to achieve maximum power load. The maximum chord length of the blade profile is located radially at r/R = 0.44, measuring 66 mm, and the maximum installation angle corresponds to r/R = 0.17, at 22.5°. The minimum chord length and minimum installation angle are both at the blade tip position.

**Figure 15:**
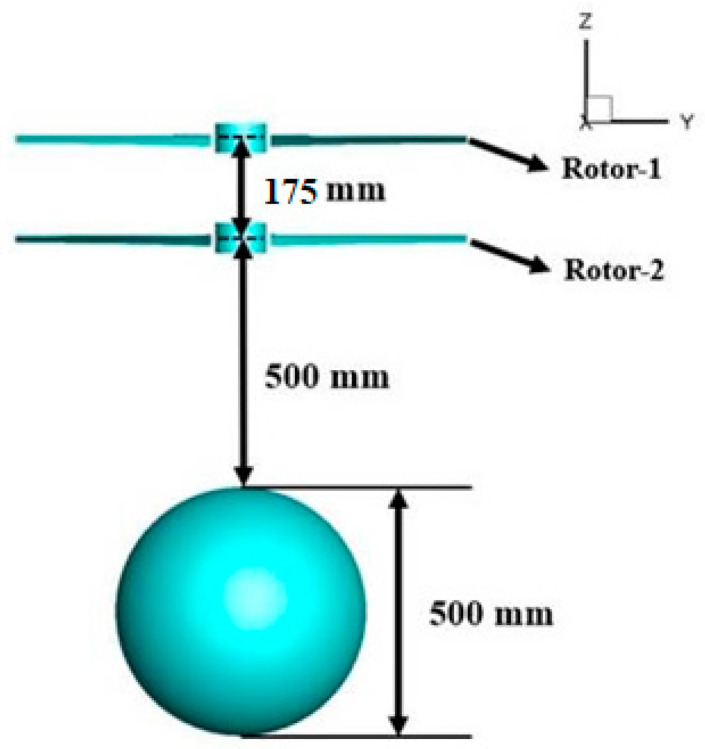
UAV coaxial-rotor balloon model.

Further integrating the requirements for balloon payload, Figure 15 illustrates the conceptual design scheme adopted for the aerodynamic design of the coaxial-rotors UAV.

#### 4.2.1. Aerodynamic Performance of a Single Rotor

**Figure 16:**
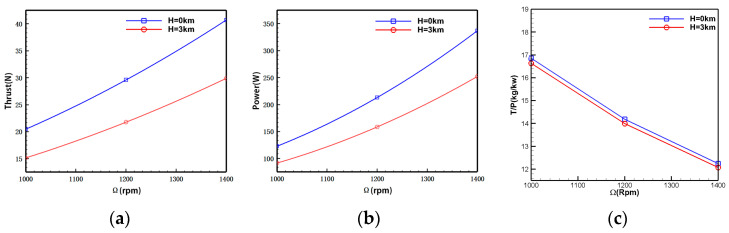
Aerodynamic performance of individual rotors (without balloon interference). (**a**) Rotor thrust-rotational speed relation. (**b**) Rotor power-rotational speed relation. (**c**) Rotor power load-rotational speed relation.

Figure 16 shows the relationship between the aerodynamic forces of individual rotors and rotational speed at different altitudes. According to the computational results, the designed rotor exhibits significantly improved performance compared to the original. At H = 0 km and 1400 rpm, the power loading reaches 12.24 kg/kW. At the same rotational speed, increasing flight altitude leads to approximately a 25.5% reduction in rotor thrust and power. Due to the rapid decrease in rotor power with increasing altitude, the variation in power loading is relatively minor.

#### 4.2.2. Aerodynamic Performance of Coaxial Rotors

**Figure 17:**
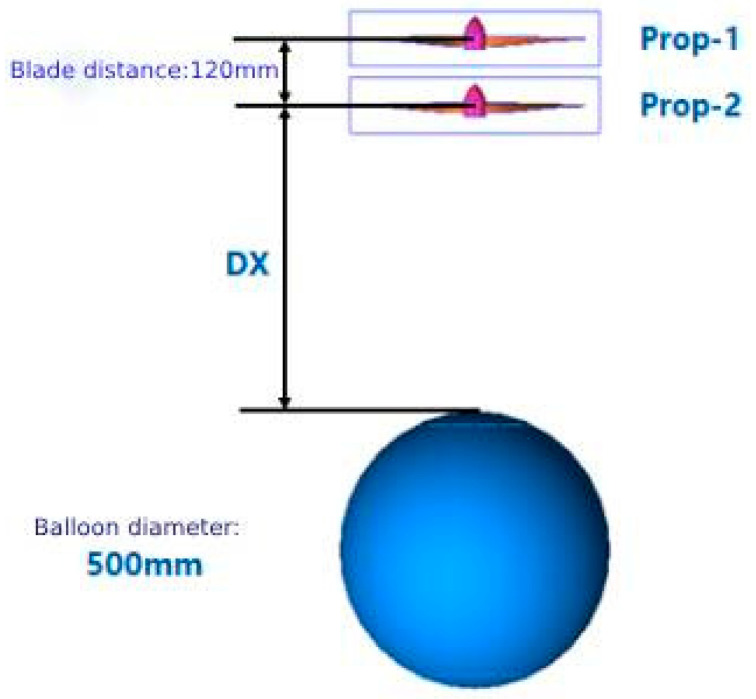
Coaxial-Rotor + Balloon Scheme.

Aerodynamic performance in the hover phase at H = 3 km altitude is analyzed for different distance ratios of coaxial rotors to balloons (Figure 17). The distance between the tip-path-planes of the upper and lower rotor is 120 mm, and the distance from the center of Prop-2 to the top of the balloon is 1.75.

#### 4.2.3. Space Ratio Effects

**Figure 18:**
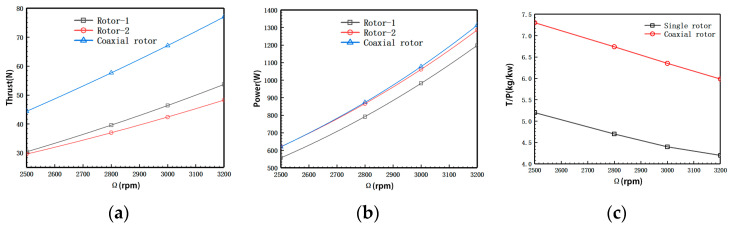
Relationship between the essential aerodynamic performance of the rotor and rotational speed. (**a**) Thrust-rotational speed relation. (**b**) Power-rotational speed relation. (**c**) Rotor power load-rotational speed relation.

Figure 18 illustrates the variation of aerodynamic forces of the initial rotor model with rotational speed. The rotor thrust, power, and rotational speed maintain a good linear variation relationship within the calculated rotational speed range. There is a more significant power load at low rotational speeds.

The calculation results show that when the axial distance of the coaxial rotors is smaller (distance ratio less than 0.3), significant aerodynamic interference occurs between Rotor 1 and Rotor 2. This interference leads to a 30% decrease in the thrust of each rotor compared to the isolated rotor conditions at the same rotational speed and a 25% reduction in the total power load of the coaxial-rotor system. It can be inferred that, within a certain range, increasing the spacing between the rotors will effectively enhance the efficiency of the coaxial-rotors system, and there exists an optimal rotor spacing [35]. 

#### 4.2.4. Design Results of the Whole Aircraft

The distance ratio is adjusted to obtain the coaxial-rotor design results. The distance ratio between the upper and lower rotors is 0.39. The distance ratio between the lower rotor and the balloon is 1.12.

**Figure 19:**
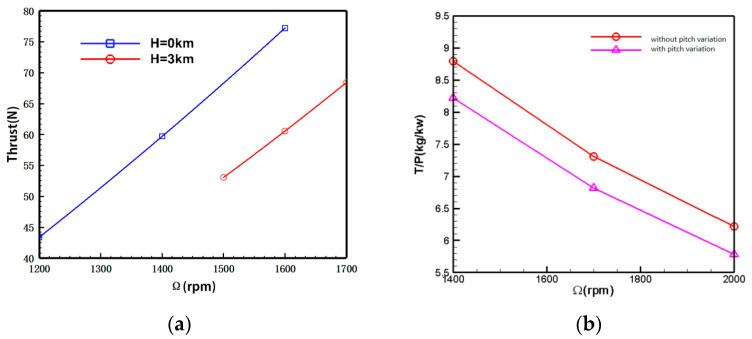
Relationship between the essential aerodynamic performance of the rotor and rotational speed. (**a**) Thrust-rotational speed relation. (**b**) Power-rotational speed relation.

Figure 19 depicts the variation of the rotor’s macroscopic aerodynamic performance with rotational speed. The computational results show that within the range of calculated rotational speeds, rotor thrust, power, and power loading all exhibit linear increases with increasing rotational speed.

**Figure 20:**
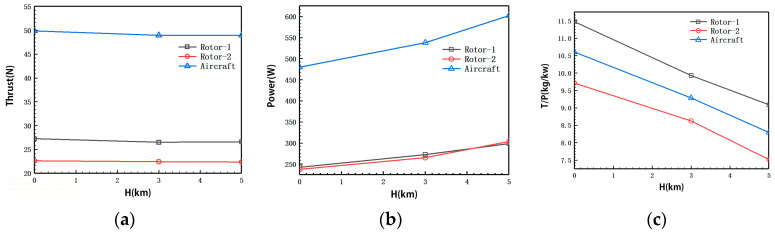
Aerodynamic calculation results of a coaxial rotors system under torque balance conditions. (**a**) Thrust-height relation. (**b**) Power-height relation; (**c**) power load-height relation.

Figure 20 presents the aerodynamic calculation results of the coaxial-rotor system under the condition of torque balance between the upper and lower rotors. To ensure torque balance within the system, the rotational speed, thrust, and power of the upper rotor are slightly higher than those of the lower rotor.

To facilitate comparison of the effects of coaxial rotors’ aerodynamic interference on blade aerodynamic performance, Table 2 compares the aerodynamic forces of the optimized individual rotors with those of the coaxial rotors. From the results of the thrust calculation, it can be seen that with the coaxial rotors distance of 175 mm, when H = 3 km and the rotational speed is 1600 rpm, the upper and lower rotor thrusts in the coaxial-rotors system decrease by 16.9% and 30.1%, respectively. The power load decreases by 21.21% compared to when the rotors are isolated. Thus, it can be seen that the aerodynamic interference of the coaxial rotors causes a significant reduction in both aerodynamic forces. Notably, the lower rotor loses about twice as much aerodynamic force as the upper rotor. Compared to a single rotor, the coaxial rotor experiences aerodynamic interference, resulting in a lower efficiency for the coaxial rotor. This difference reflects the actual efficiency disparity between the two configurations.

## 5. Aerodynamic Analysis of Coaxial-Rotors UAV

### 5.1. Variable Pitch Analysis

**Figure 21:**
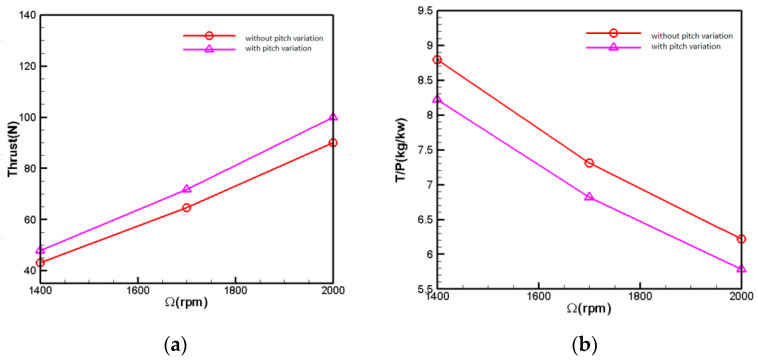
UAV Small Range Aerodynamic Comparison. (**a**) Coaxial rotors’ thrust-rotational speed relation; (**b**) coaxial rotors’ power load-rotational speed relation.

Figure 21 compares the aerodynamic forces of the coaxial rotors with and without slight pitch variation at an altitude of H = 5 km. In the figure, the pitch variation method increases the installation angle of both upper and lower rotor blades by 2°. The computational results indicate that, after pitch variation, the total rotor thrust increases by approximately 10.9%, demonstrating a noticeable effect of pitch variation. With a total thrust demand of 78 N, the rotational speed decreases by 5% after pitch variation. However, from the power loading curve perspective, it is observed that the power efficiency of the rotor blades decreases by around 7% after pitch variation compared to before the pitch variation. It can be expected that a higher pitch setting will have a higher mean lift coefficient, and a lower lift to drag ratio, thus leading to the lower efficiency.

**Figure 22:**
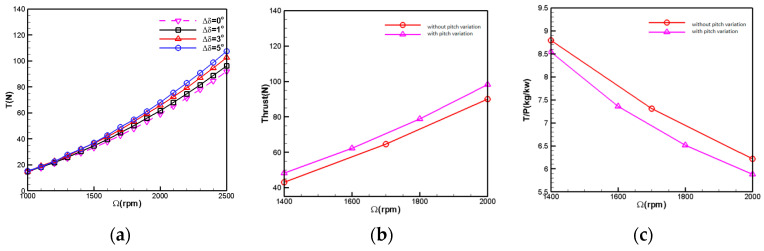
Increased pitch variation aerodynamic comparison (H = 5 km). (**a**) Change in thrust after individual rotor pitch variation; (**b**) coaxial rotors’ thrust-rotational speed relation; (**c**) coaxial rotors’ power load-rotational speed relation.

Figure 22 illustrates the aerodynamic force comparison with increased pitch variation at 5 km altitude. The pitch variation method involves increasing the installation angle of the upper rotor blades by 3° while keeping the pitch of the lower rotor blades constant. From the results of the individual rotor with pitch variation, it can be observed that, as the rotational speed increases, the effect of pitch variation becomes more pronounced. At a rotational speed of 1900 rpm, the efficiency of pitch variation results in approximately 1.5 N of additional thrust per degree increase in pitch. At 2500 rpm, a 1-degree rise in pitch results in approximately 3 N of additional thrust. The computational results from Figure 18 indicate that the total rotor thrust increases by approximately 11% after pitch variation. With a total thrust demand of 78 N, the rotor speed decreases by 3.7% after pitch variation. However, from the power loading curve perspective, the power efficiency of the rotor blades after pitch variation decreases by 3% compared to before the pitch variation.

### 5.2. Flow Field Analysis

**Figure 23:**
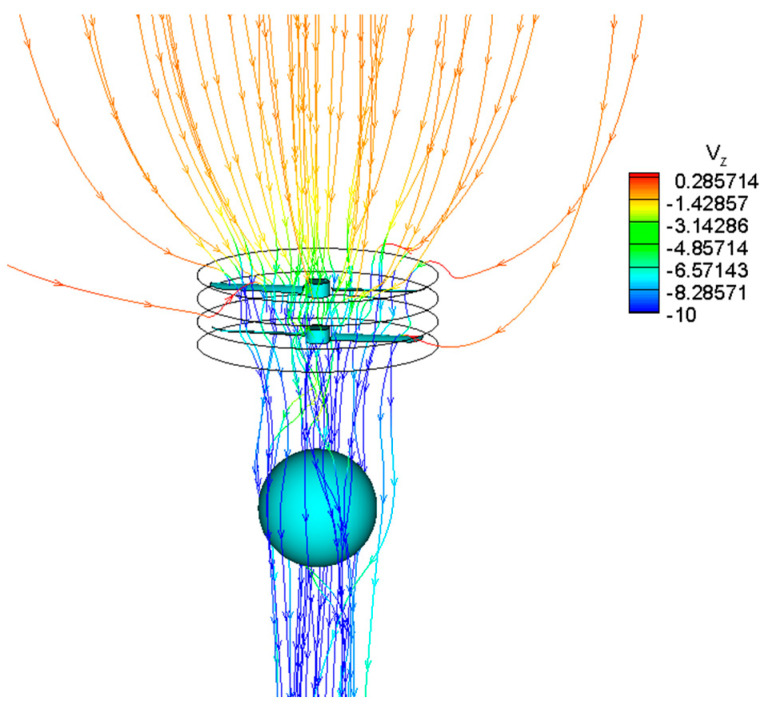
Coaxial rotors balloon 3-D spatial streamline distribution.

Figure 23 depicts the three-dimensional spatial streamlined distribution of the coaxial rotors with the balloon for the hovering state with no forward flight velocity. The rotating speed of the rotor is 1450 rpm and the altitude is 0 km. The Reynolds number based on the blade chord of the rotor feature profile (0.7 times rotor radius) is 1.90 × 10^5^. The figure shows that the rotational motion of the coaxial rotors generates a certain amount of suction effect above the rotor disk. Hence, the induced suction velocity noticeably increases as the free stream gradually approaches Rotor-1. Beneath the Rotor-2 disk, conversely, due to the injection of energy into the airflow by the rotating coaxial rotors, the slipstream velocity of the rotor further significantly increases.

**Figure 24:**
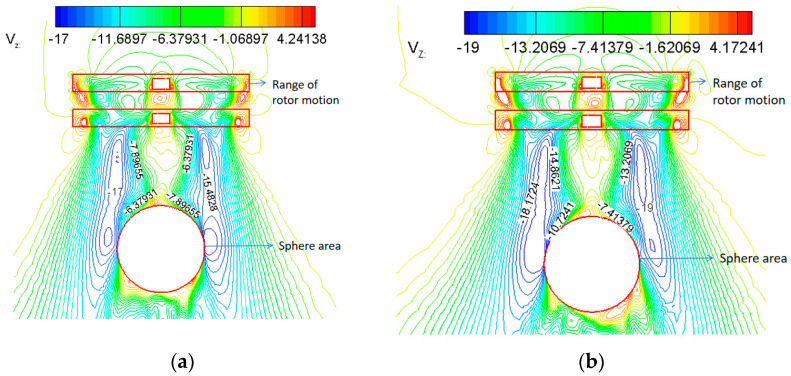
Contours of downwash velocity of a rotor on a symmetrical surface. (**a**) H = 0 km, 1450 rpm; (**b**) H = 3 km, 1680 rpm.

Figure 24 is the cloud diagram of the rotor downwash’s velocity contours at the symmetry plane’s location at different flight altitudes. Accordingly, the rotor downwash velocity reaches a maximum below the radial position r/R = 0.5 of the blades, affected by Rotor-1, Rotor-2 rotational acceleration, with a magnitude of 17 m/s at an altitude of H = 0 km. At an altitude of H = 3 km, the maximum is about 19 m/s. Near the tip of the balloon, the effect of balloon retarding causes a rapid decrease in rotor downwash velocity but is still subject to a 6 m/s~10.7 m/s rotor downwash.

**Figure 25:**
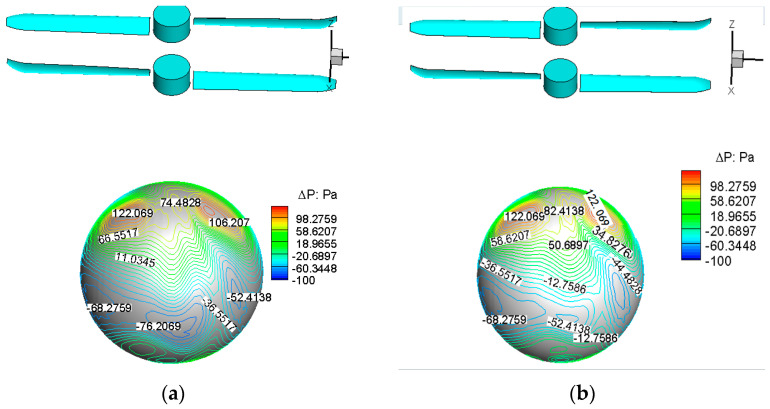
Incremental pressure distribution on balloon surface under the influence of rotor downloading. (**a**) H = 0 km, 1450 rpm; (**b**) H = 3 km, 1680 rpm.

Figure 25 further illustrates the pressure increment distribution contour map on the balloon’s surface under the influence of the rotor downloading. From the computational results emerges the observation that the blocking effect of the balloon on the high-speed rotor downloading leads to a significant increase in pressure near the top of the balloon. This effect is notable for a maximum increment of up to 122 Pa. Since the pressure at the top of the balloon is significantly higher than at the bottom, the rotor downloading generates an additional drag force of approximately 6 N on the balloon.

**Figure 26:**
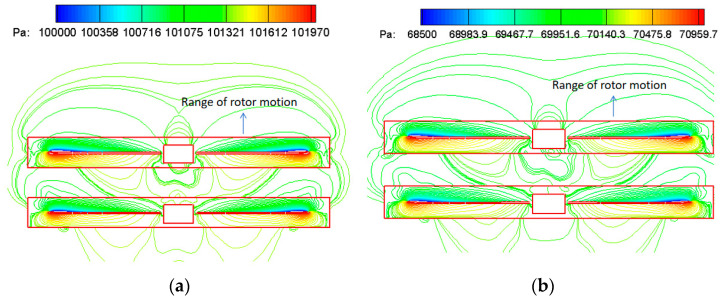
Coaxial rotors pressure distribution at the location of the symmetry plane at different altitudes. (**a**) H = 0 km, 1400 rpm; (**b**) H = 3 km, 1700 rpm.

Figure 26 depicts the pressure distribution contour map at the symmetric plane location of the coaxial rotors at different altitudes. The computational results indicate that the thrust of the coaxial rotors primarily originates from the outer blade sections. However, due to their proximity, the high-pressure region on the lower surface of Rotor-1 mixes with the low-pressure area on the upper surface of Rotor-2. This mixing decreases the thrust of both rotors under the aerodynamic interference of the coaxial-rotor configuration. As a result, the individual thrust of the upper or lower rotor is less than that of an isolated rotor.

**Figure 27:**
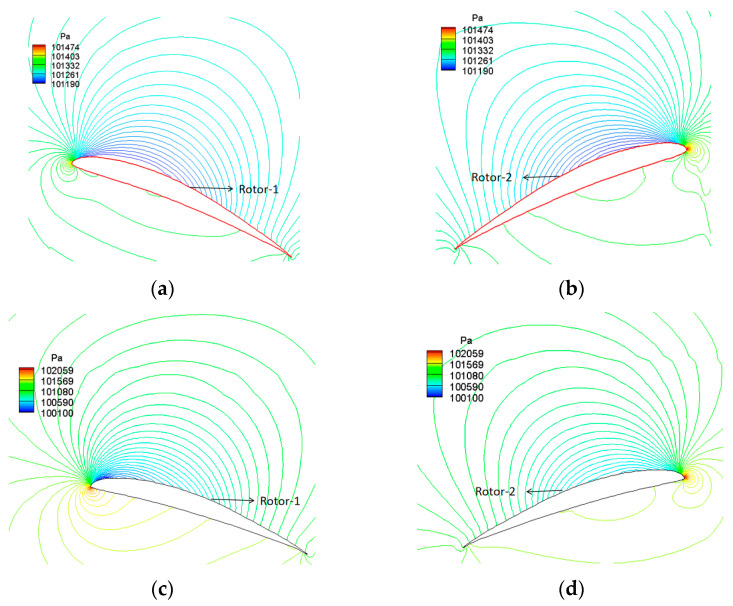
Comparison of pressure distribution in different radial position profiles of coaxial rotors (H = 0 km, 1400 rpm). (**a**) r/R = 0.22, Rotor-1; (**b**) r/R = 0.22, Rotor-2; (**c**) r/R = 0.78, Rotor-1; (**d**) r/R = 0.78, Rotor-2.

Figure 27 further contrasts the pressure distribution across the chord section at different radial positions of the coaxial rotors. As the coaxial rotors are coaxial and counter-rotating, the high-speed axial slipstream induced by Rotor-1 on Rotor-2 somewhat reduces its effective angle of attack. This effect is particularly noticeable for the outer wing section, where leading-edge stagnation occurs visibly on the airfoil’s upper surface. Consequently, the aerodynamic performance of Rotor-2 is inferior to that of Rotor-1.

**Figure 28:**
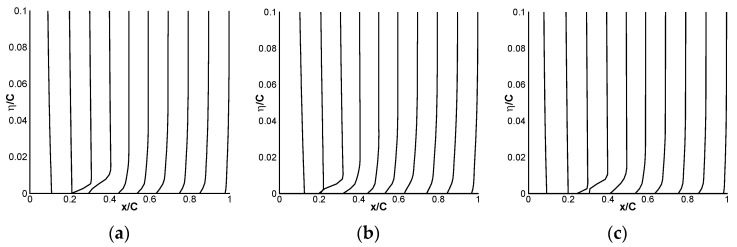
Schematic diagram of the velocity profile at the 99% blade tip position. (**a**) x/c = 0.2; (**b**) x/c = 0.5; (**c**) x/c = 0.9.

Figure 28 shows the velocity profile schematic of the mean flow at the 99% blade tip position along the chordwise direction. Due to the influence of the blade tip vortex, flow separation may occur on the rear part of the airfoil along the chordwise direction. In fact, flow separation does not occur on the rotor surface within 78% of the blade radius.

### 5.3. Verification Measurement

**Figure 29:**
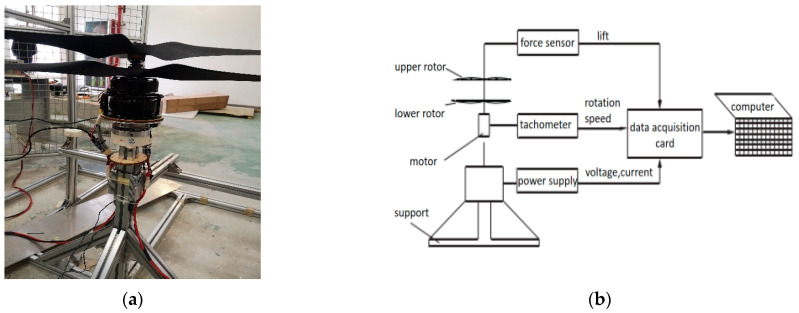
The schematic of the motor/rotor experiment bench and test principle. (**a**) The experiment bench. (**b**) Workflow diagram.

The selected experimental motor model is the UA90 KV100 motor. The experiment adopts a basic testing system [36,37]. The schematic of the experiment bench and test principle of the coaxial rotors are illustrated in Figure 29.

**Figure 30:**
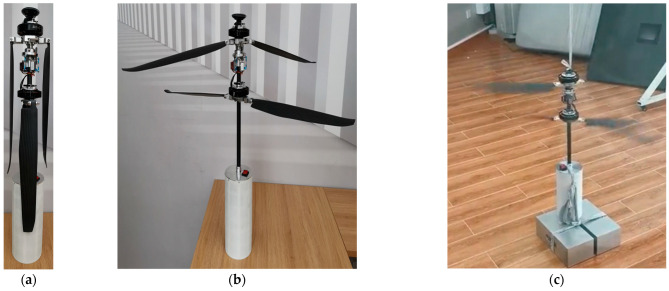
Schematic of calibration testing for coaxial dual-rotor balloon in non-deployed state. (**a**) Rotor folded state. (**b**) Rotor deployed state; (**c**) Rotation state (vibration reduction calibration).

The test environment is as shown in Figure 30, demonstrating the use of internally developed testing equipment and environment.

**Figure 31:**
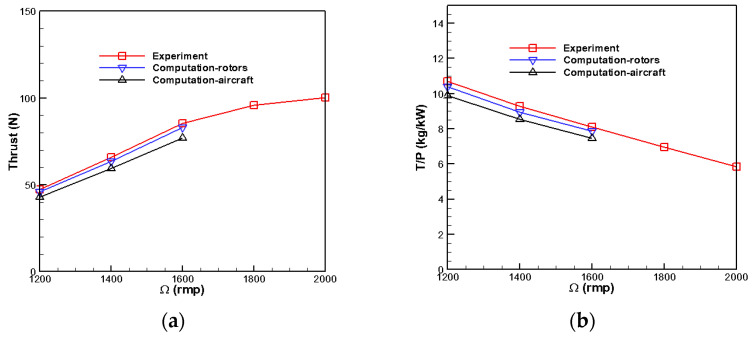
Comparison of experimental measurement and calculation results. (**a**) Thrust-throttle relation. (**b**) Proportional efficiency of thrust-power.

Figure 31 shows the experiment results. Comparing the calculations and experiments reveals that the experimental results agree with the coaxial-rotor system’s calculation results. However, due to the balloon’s drag, there is a specific deviation between the full aircraft calculation results and the test without the balloon.

For the thrust demand 78 N, a single rotor should generate a thrust of at least 4 DaN. At an altitude of H = 0 km, the rotation speed is at least 1500 rpm. At 3 km and 5 km altitudes, higher rotation speeds and throttle inputs are required. It can be seen that choosing the UA90 KV100 motor and optimized coaxial rotors is reasonable. They can ensure the power system has a throttle margin for complete aircraft.The single rotor partial test results are shown in Table 3.

## 6. Conclusions

(1) The bionic conceptual design is excellent. It can fully integrate Chinese parasol seed flight features and UAV flight requirements.
(2) The UAV, bionically modeled as a Chinese parasol seed, has high essential aerodynamic performance. Its navigation stability and variable pitch flight capability are good. The cruise power load reaches 8.36 kg/kw, and the cruise flying thrust force is not less than 78 N at coaxial-rotor and rotor-balloon distance ratios of 0.39 and 1.12, respectively. Despite achieving a cruise power loading of 10.61 kg/kw, the cruise flying thrust force of the single rotor is only 39 N under these conditions. Therefore, the bionic simulation of the coaxial-rotor configuration is superior to the single rotor configuration.
(3) The UAV has the “blocks stability phenomenon” formed by decreased rotor downwash speed and the balloon’s additional negative pressure. However, it can provide space for omnidirectional detection and reconnaissance missions.
(4) The efficiency experiment and simulation of the coaxial rotors/motor can be agreed upon. The present method and the bionic configuration provide a feasible design and analysis strategy for coaxial-rotor UAVs.

One of the potential challenges in the design of bionic conceptual design on UAVs is the issue of fluid-structure interaction (FSI), particularly the accuracy of numerical simulations for FSI involving flexible structures. Additionally, the complexity of manufacturing due to bionic structures presents further difficulties. This paper employs the MRF model method combined with structured-unstructured hybrid mesh techniques and utilizes the SST turbulence model to solve for numerical simulations. Using different materials for 3D printing, we addressed the manufacturing challenges of complex surfaces in biomimetic structures. Additionally, this reduces manufacturing costs compared to traditional machining methods.

## Figures and Tables

**Table 1:** Comparison of calculated results of rotor thrust coefficient.

Rotation Speed (RMP)	Experiment	CFD		
		Coarse	Middle	Fine
1750	0.00455	0.004857	0.00474	0.00469
2250	0.00462	0.004998	0.00491	0.00487

**Table 2:** Aerodynamic comparison of individual rotor with coaxial rotors.

Height	Rotation Speed	Single Rotor Thrust	Upper Rotor Thrust	Lower Rotor Thrust	Single Rotor Efficiency	Coaxial Rotors Efficiency
3 km	1600 rpm	39.34 N	32.70 N	27.49 N	10.61 kg/kw	8.36 kg/kw

**Table 3:** Single rotor partial test results.

Ω (RPM)	Thrust (N)	(g/W)
1200	42.91	9.88
1400	59.32	8.55
1600	76.99	7.46
3505	172.38	6.71
3700	185.02	6.21

## Data Availability

The authors do not have permission to share data.
